# Facts and Acts Worth Knowing

**Published:** 1895-06

**Authors:** 


### FACTS AND ACTS WORTH KNOWING.

The Ætna Live Stock Insurance Co., a mutual assessment company of Pennsylvania with headquarters at Philadelphia, bids fair to end up in the courts. Numerous lawsuits against policy-holders and the counter-suits of the latter against the company are now in process of solution. The Insurance Commissioner of the State has been asked to investigate the management.

The Missouri Board of Agriculture finds it a profitable investment to employ from time to time the services of veterinarians to lecture before their Farmers' Institutes on subjects which interest and instruct owners and breeders of live-stock, a plan that might be employed profitably by the Boards of every State.

The establishment of a veterinary college for Ireland is under consideration in Great Britain, and is likely to be accomplished.

The City Government of Philadelphia has not been slow to recognize the value of true veterinary service, hence the employment of the following corps of veterinarians: Consulting Veterinary Surgeon to the Meat Inspection Department under the Department of Public Safety, Dr. Leonard Pearson; Chief Inspector, Dr. A. F. Schrieber; Milk Inspectors under the direction of the City Board of Health, Drs. G. R. Hartman, Frank L. Smith, Chas. M. Earnest; City Veterinarian to the Fire and Police Department horses, Dr. John R. Hart.

The Keystone Veterinary Medical Association of Philadelphia and vicinity, at their April meeting, strongly urged the defeat of those bills pending in the State Legislature destined to prevent the entrance of Western dressed-beef products, except under such State inspection that would make the traffic almost prohibitory. Such selfish laws are often great blows to those who promote them and result in material injury to the States that enact them.

Prof. H. C. Wood, of the Medical Department of the University of Pennsylvania, was somewhat but agreeably surprised to find that some six members of the Veterinary Department's graduating class won the perfection mark of 100 in their examinations under him. This is a record hardly equalled in any former year by any class of the different departments of medicine.

At the annual meeting of the Board of Trustees of the American Veterinary College, held at the office of President Weisse of the Board, in the City of New York, on Tuesday evening, May 14, 1895, a hearing was accorded representatives Drs. W. L. Rhoads, H. D. Hanson, Bryce Mars, and W. Horace Hoskins, of the Alumni Association, who pleaded for increased representation of the latter body on the Board of Trustees. After mature deliberation the Board granted the request, and it will be the province of the Association at their next regular meeting to elect the additional representatives granted. We believe that it is always a wise policy to concede a generous representation on such Boards to those who are most directly and deeply interested, and none can have their alma mater's welfare more at heart than her own children.

The address of Dr. Austin Peters before the *Ploughman's* Farmer meeting in Boston on April 6th was an unusually fair, concise, and conservative review of the question of tuberculosis in its present aspect and the knowledge we possess. Dr. Peters speaks with authority, for he has been one of the most earnest, conscientious searchers after the truth and light on this subject for many years, and has had a wide field of observation at his command. We therefore feel that no apology is needed in opening wide our columns to quote from his address on this occasion.

The Veterinary Department of the University of Pennsylvania grants twelve free scholarships to the State and three to the city of Philadelphia, through the generosity of the State and city in aiding the school by grants of money and land for college purposes.

In conjunction with the valuable course of lectures now being given to the horseshoers of Philadelphia, many of the masters as well as journeymen are pursuing a special course of anatomy, with dissections, at the Veterinary Department of the University of Pennsylvania. This means much for the welfare of the shoeing art in Philadelphia.

Dr. D. LeMay has in French press at Montreal, Canada, a history of the recent false report of an outbreak of contagious pleuro-pneumonia in Kansas.

The value and need of the recently proposed law in California establishing State and County Veterinarians awakened much comment among the veterinarians and wide differences of opinion as to the wisdom of such an act at this time.

Rivalry between two of Cincinnati's veterinarians for the place of veterinarian to the recent dog-show ended in a disgraceful street encounter which will have its termination in the courts.

The resolutions recently adopted by the California State Veterinary Medical Association, condemnatory of Dr. T. Carpenter, of Oakland, for aiding in the defeat of certain State legislation fathered by the Association, has culminated in a libel suit against Secretary Archibald.

Rabies among the canines is said to prevail in epizootic form throughout parts of Great Britain.

Paris conducts a dog-market on Sunday for the sale and exchange of canines.

One of Washington's veterinarians will in the early future erect there a thoroughly modern and well-equipped hospital for members of the canine and feline families.

Red water, or Texas fever, has broken out in Northern Australia. It is thought to have reached there through cattle from Queensland brought from the northwest territory.

The museum of the National Veterinary College has been enriched by the presentation of a corroded silver-plated table-knife recently found in the stomach of a cow.

Loss and gain in the weight of horses vary much from day to day. A single drive has been noted to cause the loss of eighteen pounds, while an apparent gain of sixty pounds has been noted under ordinary conditions in three days.

The American Veterinary Hospital of Allegheny, Pa., which opened April 1, with free consultation from 5 to 9 A. M. and 6 to 9 P. M., has been closed by the constable. Dr. Wall, the head of the institution, is now confined in jail waiting trial on the charge of perjury. The hospital staff, while claiming to be veterinary surgeons, were not graduates of any veterinary college.

Western Pennsylvania veterinarians deserve much credit for their earnest efforts in prosecuting violators of the law regulating the practice of veterinary science in Pennsylvania. In both cases the violators promised to cease violating the statute if leniency was shown in their sentences. Drs. Martin and Falconer should keep up the good work; it will teach a wholesome lesson.

Horseflesh is being manufactured into an animal food for chickens.

Parisians are finding it more profitable to buy horses in the United States than at home. Recently some two hundred horses were purchased in Chicago for one exportation.

Connecticut has passed a law making the docking of horses' tails a misdemeanor.

